# Climate change and California’s terrestrial biodiversity

**DOI:** 10.1073/pnas.2310074121

**Published:** 2024-07-29

**Authors:** Susan Harrison, Janet Franklin, Rebecca R. Hernandez, Makihiko Ikegami, Hugh D. Safford, James H. Thorne

**Affiliations:** ^a^Department of Environmental Science and Policy, University of California, Davis, CA95616; ^b^Department of Geography, San Diego State University, San Diego, CA92182; ^c^Department of Land, Air and Water Resources, University of California, Davis, CA95616; ^d^Wild Energy Center, University of California, Davis, CA95616; ^e^National Institute for Environmental Studies, Tsukuba305-8506, Japan; ^f^Vibrant Planet, Incline Village, NV89451

**Keywords:** California, terrestrial, biodiversity, climate change, solar energy

## Abstract

In this review and synthesis, we argue that California is an important test case for the nation and world because terrestrial biodiversity is very high, present and anticipated threats to biodiversity from climate change and other interacting stressors are severe, and innovative approaches to protecting biodiversity in the context of climate change are being developed and tested. We first review salient dimensions of California’s terrestrial physical, biological, and human diversity. Next, we examine four facets of the threat to their sustainability of these dimensions posed by climate change: direct impacts, illustrated by a new analysis of shifting diversity hotspots for plants; interactive effects involving invasive species, land-use change, and other stressors; the impacts of changing fire regimes; and the impacts of land-based renewable energy development. We examine recent policy responses in each of these areas, representing attempts to better protect biodiversity while advancing climate adaptation and mitigation. We conclude that California’s ambitious 30 × 30 Initiative and its efforts to harmonize biodiversity conservation with renewable energy development are important areas of progress. Adapting traditional suppression-oriented fire policies to the reality of new fire regimes is an area in which much progress remains to be made.

## 1. California’s Diversity

“If California were a country…” often leads into a comparison of this single United States state to the rest of the world. California (~424,000 km^2^; spanning >10° latitude and longitude and 4,400 m elevation) has more native plants, animals, and vegetation types; more variation in bedrock and soils, climate, and fire ecology; and superimposed on this natural mosaic, a larger human population, larger economy, more diverse agriculture, more invasive species, and more renewable energy production than any other US state or most entire countries. Home to some of the earliest environmental laws and movements, California continues to innovate in both climate adaptation and biodiversity protection. Here we argue that California is a national and world leader in the magnitude and complexity of the threats that climate change poses to terrestrial biodiversity, and also a leader in using an understanding of these threats to shape new approaches. We set the stage for our review and synthesis by examining key aspects of California’s terrestrial diversity (physical, biological, human). We next review the major types of threat posed by climate change (direct, interactive, fire-related, and related to land-intensive renewable energy development), and we illustrate the direct effects with a novel analysis of shifting biodiversity hotspots. We conclude by considering recent policy responses that attempt to protect terrestrial biodiversity in the context of the different types of threats and offering recommendations.

Our Perspective addresses a main theme of the Special Feature: mitigation and adaptation to the current and future impacts of climate change on California’s environmental, social, and economic sustainability (1). Sustainability is a concept well known for its multidimensionality (e.g., ref. 2); rather than the classic economic concept of sustainability, which centers on the maintenance of human uses of natural resources and ecosystem services, our focus is on sustainability in the sense of United Nations Sustainable Development Goal 15—Life on Land, which emphasizes the maintenance of biodiversity in the face of human impacts as a critically important end in itself. We consider the multiple threats that climate change poses to California’s terrestrial ecosystems, as well as the policies aimed at mitigating these threats and sustaining our biological legacy.

### 1.1. Physical Diversity.

The template for California’s biological diversity is its diversity of earth materials, forms, and processes (“geodiversity”; 3). A Mediterranean climate with its cool wet winters and hot dry summers is fundamental to understanding California’s terrestrial biodiversity (4). Found in 70% of California and in four other world locations, this climate with its short growing season and long fire-prone season generates the highest levels of plant diversity and endemism outside the tropics (5–7). Evergreen shrublands are its most characteristic vegetation, but forests, wetlands (8), woodlands, and grasslands are extensive and varied. The other third of California comprises the interior warm deserts in the southeast and cold Great Basin deserts in the east. The state’s climate encompasses coastal-to-interior, latitudinal, and elevational gradients that produce enormous variation, such as the range in mean annual precipitation from >300 cm in redwood forests to <5 cm in the southern deserts. California supports the highest interannual variability in precipitation in the United States, with episodic atmospheric rivers (i.e., moisture-laden flowing columns of the atmosphere) contributing 30 to 45% of California’s annual precipitation, and multiyear droughts—which have major influence on wildfire occurrence—occurring with regularity (9).

Relatively recent (late Miocene to Pleistocene) uplift of the state’s mountains exposed diverse bedrock types, weathering in variable settings to produce a vast number of named soil types differing in texture, chemistry, and water availability (10). These soils support hundreds of distinct vegetation types (435 Alliances, >1,200 Associations; 11, 12). Geologic and topographic diversity alone is not enough to generate high biotic diversity, as can be seen by comparing California with lower-diversity Alaska (four times larger in area and also with dramatic topography of tectonic origins); the Mediterranean climate plays a decisive role in evoking high biodiversity from sharp physical gradients (7).

Fire is the most important natural disturbance regime shaping ecosystem dynamics in California’s upland habitats with a Mediterranean climate (13). Most of California is intrinsically fire-prone due to moderate to high plant productivity combined with a lengthy dry season. However, ignitions prior to European settlement only occurred where lightning coincided with dry weather or where Indigenous burning. Fire is critically important in California, yet its historical frequency, severity, and ecological effects are complex and poorly understood (14, 15).

### 1.2. Biotic Diversity.

California supports 4,266 full species of naturally occurring (native) plants, 1,307 (31%) of which are endemic; these numbers change to 5,006 and 1,846 (37%) when considering the California Floristic Province (CFP, >322,000 km^2^; 87% in California; 16, 17), the biotic region defined by the Mediterranean climate that excludes the desert but includes small parts of Oregon and Baja California. Most CFP endemics or near-endemics are either shrubs with sclerophyllous (hard) evergreen leaves, or herbaceous plants with bulbs or seeds capable of belowground persistence, as is characteristic of the Mediterranean biome. More temperate life forms, such as many broadleaved deciduous trees, include few Californian endemics and are found in wetter locations such as coastal and riparian areas and north-facing slopes (oaks being a notable exception). This flora is believed to have originated via persistence of subtropical lineages from a wetter Eocene, incursion of temperate and desert-adapted lineages during and after the drying Miocene, and rapid Plio-Pleistocene radiation by some arid-adapted shrub and herb lineages to produce endemic species (18, 19). The Miocene onset of widespread fire also played a critical role in plant speciation and adaptation (20).

The state’s diversity of native terrestrial mammals (163 species), birds (>300 breeding species), reptiles and amphibians (171 species), insects (ca. 30,000 species), and other animals is high, as expected from its large size and ecological variability (21). However, Californian animal endemism is not high in most groups, except for a few notable for their low mobility (e.g., *Ensatina* legless salamanders; various flightless arthropods; 7). Even its most famous near-endemic animal, the condor (*Gymnogyps californianus*), had a broader distribution until recently (22).

Invasive species are more numerous in California than in any other state, including ca. 1,300 plant species, and continue to accumulate. California’s invasibility is attributed largely to its island-like evolutionary history of isolation, followed by the influx of humans and their associated biota from climatically similar parts of Eurasia and northern Africa. Invasive grasses have been especially impactful as they have transformed perennial grasslands into annual ones with reduced biodiversity (23).

### 1.3. Human and Institutional Diversity.

California was one of the most linguistically diverse places on earth, with at least 78 languages among its hundreds of thousands of Indigenous inhabitants. Small-scale communities predominated, and human–environment relationships were highly diverse owing to varied habitats and resources (24). Today, the state’s Native American population is the most urbanized in the country, with relatively few Tribal communities living on their traditional lands (24), and those who do live on these lands rarely possess them. A recent focus on recovering and understanding traditional ecological knowledge is improving clarity about historical and contemporary Indigenous influences, respectively, on California ecosystems, which promote viable populations of culturally important plants and animals through active land tending (pruning, propagation, cultural burning; see ref. 25), (24) but much uncertainty still exists as to the intensity and geographic extent of those influences (15). The present-day human population of California is heavily coastal and urban (26), and has the greatest ethnic diversity and percentage of immigrants in the nation. Interior valleys have been transformed by the most diverse agriculture in the world (25), with >400 cash crops, but the farming population is sparse.

California has a history and diversity of environmental institutions, agencies, policies, and laws addressing nature conservation as well as climate change. The state is home to some of the nation’s first National Parks (Yosemite, 1864), State Parks (Big Basin, 1927), forest reserves (San Gabriel and San Bernardino, 1892 to 1893), environmental organizations (Sierra Club, 1892), and Land Trusts (Marin Agricultural Land Trust, 1980). An astonishing 46% of the state’s area is park or wilderness land, mainly in mountains and deserts (27, 28). Twentieth-century landmarks included the California Environmental Quality Act (1970; amended to regulate greenhouse gasses, 2007), Coastal Protection Act (1972), Natural Communities Conservation Act (1991), Global Warming Solutions Act (2006), Sustainable Communities and Climate Protection Act (2008), and California’s 30 × 30 Initiative (2020). Innovative approaches to biodiversity conservation, such as easements, mitigation banks, carbon credits, and advance mitigation of highway impacts, have been developed or improved in California (28).

For several decades, California has led the nation in laws and policies to mitigate climate change, beginning with the Global Warming Solutions Act of 2006, which set goals for the reduction of statewide greenhouse gas emissions, and the Sustainable Communities and Climate Protection Act of 2008, which set strategies for reducing greenhouse emissions from passenger vehicles (see ref. 1). Climate policies aimed specifically at the protection of biodiversity, among other goals, began with the 2007 amendments to the California Environmental Quality Act requiring that reviews of new projects consider the impacts of greenhouse gas emissions and climate change on environmental values. California has also been conducting statewide climate change assessments since 2005 and is currently undertaking its fifth assessment. These assessments provide updated spatial data from global climate models, technical reports on topics across a wide range of topics, and, more recently, regional reports intended to support local efforts to plan for, adapt and mitigate climate change.

We next turn to four of the most acute aspects of climate change’s impact on California’s terrestrial biodiversity: direct impacts, which we summarize with a new analysis of projected shifts in biodiversity hotspots; interactions of climate change with other global change drivers; impacts driven by altered fire regimes; and impacts driven by climate change mitigation efforts, particularly land-intensive solar energy development.

## 2. Direct Impacts of Climate on Terrestrial Biodiversity

California’s climate has become warmer, effectively drier, and more variable since the mid-20th century and is projected to change further in the future with increasing extreme weather events (29). Climate change impacts are already observable in plant and animal species distributions in California (30, 31), including widespread tree mortality from a combination of drought, wildfire, and expansion of pests and pathogens (32), upslope shifts in plant ranges (33), retraction of lower-elevation coniferous forests (34), shifts toward smaller trees and a higher predominance of broadleaved trees relative to conifers (35), and a wide variety of changes in plant and animal distributions and abundances (36–40). Most of California’s ecosystems are water-limited rather than energy-limited (41), and the decreased soil moisture availability caused by warming in such a context may generally drive decreases in the diversity of plant communities (42, 43). Tracking their climate-driven range shifts in California’s highly complex physiography could require movement in different directions by individual species (44).

One way to seek generalities when forecasting the potential future impacts of 21st-century anthropogenic climate warming on California’s biodiversity is to consider the state’s geographic concentrations of high numbers of native and endemic plant species, which we term regional hotspots. By identifying general patterns of change in the locations of these regional hotspots, it is possible to reduce the complexity of anticipated future change, the potential limitations of current conservation approaches, and the variety of strategies that will likely be needed (36).

We present an example of such a forecast to illustrate anticipated impacts on regional hotspots (rather than individual species). We analyzed projected floristic change in the Mediterranean climate (California Floristic Province, CFP) part of California, which comprises 70% of the state and does not include the deserts; the CFP is characterized by its globally outstanding plant diversity and endemism and is well documented with georeferenced floristic data.

Briefly, we used species distribution modeling of 6,418 plant taxa (species, subspecies, and varieties) native to the Californian part of the CFP to identify current (1960 to 1990) regional biodiversity hotspots (*SI Appendix*, *Materials and Methods*). For each 20.25 km^2^ grid cell, we combined species richness with range-size-weighted rarity value for each species present to create an index of conservation value. We selected clusters of 11 or more grid cells with the top 2% of this index to define current regional hotspots in California. We next projected species’ ranges with 2061 to 2080 climate predictions from 14 global climate models (GCM)—all under the Representative Concentration Pathway (RCP) 8.5 greenhouse gas emissions scenario—using the species distribution models and again calculated conservation value. Spatial changes in regional biodiversity hotspot locations over time were based on the mean values of the conservation indices from 14 projections.

**Fig. 1.**
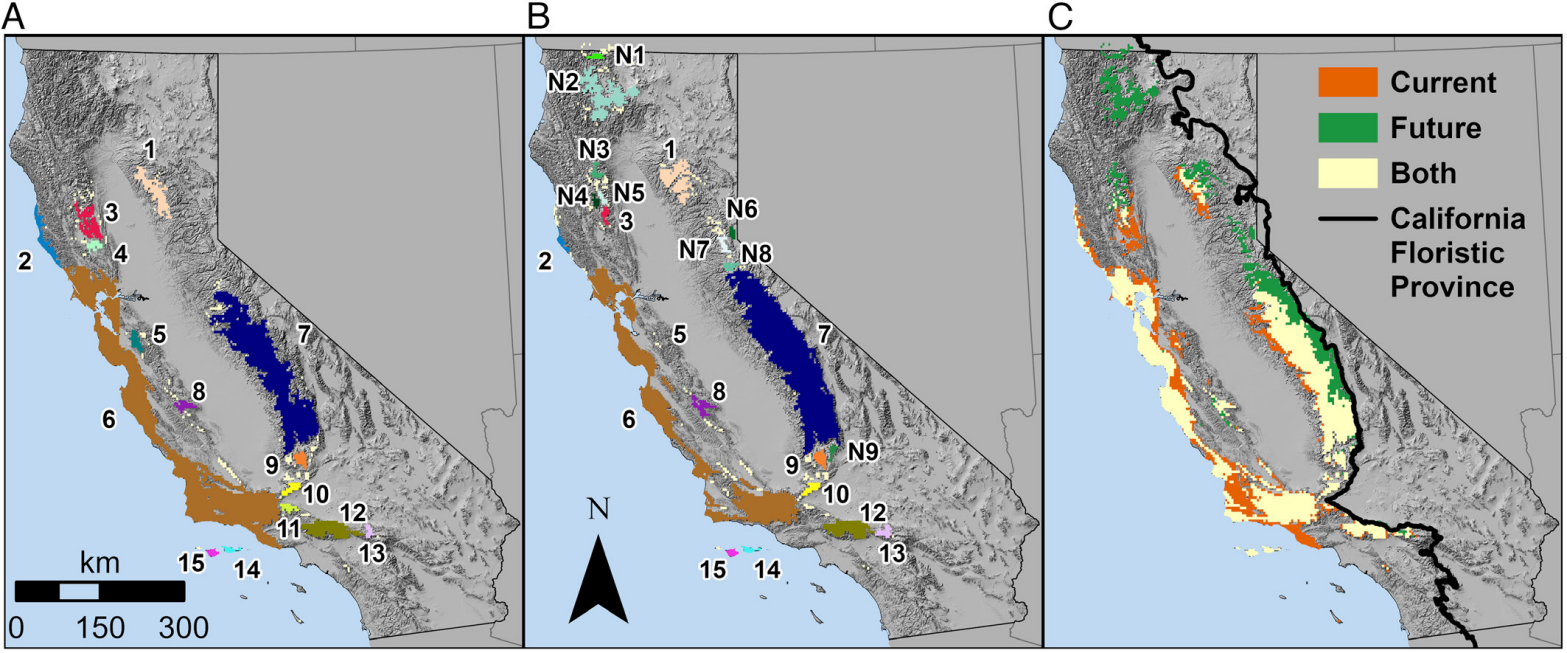
Locations of current and future regional hotspots, represented as those identified by an ensemble from 14 GCMs. (*A*) Locations of regional hotspots, labeled 1 to 15, identified under the baseline time period of 1960 to 1990. (*B*) Regional hotspot locations identified in the 2061 to 2080 time period, including those remaining from baseline, which retain their numbers from panel (*A*), and nine new locations, labeled N1 to N9. (*C*) Change in regional hotspot locations.

Our analysis identified 15 well-known current regional hotspots (*SI Appendix*, Table S1 and Fig. 1*A*), ranging from small ones such as the Channel Islands to very large areas including the Southern Sierra Nevada and the Central and Southern Coast Ranges. Under projected future climates, the 15 regional hotspots lose 18.8% of their component native species (*SI Appendix*, Table S1 ). We identified five major spatial modes of change in hotspots under future climates: i) Four current hotspots in the Channel Islands, Transverse Ranges, and Southern Coast Ranges remain relatively stable in location and were projected to lose less than half their area. ii) Three current hotspots contract by more than half their area. For example, the Central and Southern Coastal Ranges hotspot (Fig. 1) contracts and forms seven smaller coastal hotspots, possibly tracking a diminishing maritime influence. The Inner Coast Range hotspot disappears completely. iii) Three current hotspots shrink so much that they are no longer detected with this approach. iv) Five hotspots shift gradually to track suitable climate. For example, Sierra Nevadan regional hotspots tend to lose lower elevation areas and either contract or advance toward higher elevations (Fig. 1). v) Nine new hotspots emerge in northerly and/or high-elevation locations. Most of these are small, newly climatically suitable areas appearing by 2080 not far from their current locations (*SI Appendix*, Table S2 and Fig. 1).

The various spatial modes of regional hotspot dynamics suggest different general strategies for climate-adaptive conservation planning. For the hotspots that shift gradually upslope, preserving connecting habitats may be a suitable conservation strategy (45). For contracting hotspots such as those in the central coast, protection of remnant populations and refugia (46–48) could be the only available conservation strategy, especially given the additional challenges urbanization poses. For novel hotspots, a combination of land protection and assisted migration (e.g., ref. 49) could help enhance the value of these locations to protect future biodiversity.

In summary, recently observed and predicted climate-driven biotic changes in California, including those predicted by our simple model, highlight a much higher degree of complexity than the canonical poleward and upward shifts. Of course, all models have limitations, and ours did not include details found in many models of individual species (e.g., demography, dispersal capacity, functional traits, soils; 50–53). The greatest limitation of any predictive species distribution model arises from the complex interactions between climate and other factors (54). We consider some of the most critical of these interactions in the next section.

## 3. Interactive and Ecosystem-Level Impacts of Climate on Biodiversity

Climate change interacts with other land-use and land-cover changes, biotic disruptions (invasive species, pests, and pathogens), disturbances (fire, floods), and pollution, to affect terrestrial biodiversity (54). The same complex physiography that shapes California’s biodiversity also powerfully influences these additional stressors (55, 56). Urban development is concentrated in coastal areas, intensive agriculture in the Central Valley, and ranching in the foothill oak savannas; montane conifer forests have been extensively altered by logging and fire suppression (57, 58). Even desert regions have experienced urban and agricultural expansion, novel fire regimes driven by invasive plants (59), and large-scale renewable energy development (Section 5). One demonstration of interactive effects of multiple stressors comes from >40 y of surveys indicating that both land-use and land-cover change and climate change have altered butterfly distributions and diversity (60, 61); low-elevation species have declined, consistent with intensive land use, while montane species have shifted to higher elevations, consistent with warming Over the past century in California’s Central Valley, many bird species declined, and some more generalized species increased under the combined impacts of altered water availability, wetland loss and agricultural conversion (62).

Models of expected land-use change, based on population growth projections and the socioeconomic pathways used in climate forecasting, forecast severe negative impacts on the wildlife of California’s oak woodlands, savannas, and grasslands (63). When these models also consider climate change, they show climate and land-use impacts can be of the same order of magnitude, although not always in the same places (64–69). These conclusions are based on species distribution modeling that projects shifts in the distribution of climatically suitable habitat, both separately and combined with expected land-use change. Projected habitat expansion due to climate change is sometimes partially or entirely canceled by land-use change, for example for the plants in southern California (67) and the birds of oak woodlands in the Sierra Nevada foothills (66). In highly urbanized southern California, expansion of urban development poses a more immediate threat than climate change, and it also exacerbates the threat of climate change by reducing habitat connectivity (64, 65). The most effective solution to the threat of interacting urban growth and climate change, as identified by a statewide modeling study, is urban infill—pushing new urban development into the existing footprint of current cities. This strategy outperforms alternatives of protecting agricultural lands least at risk to climate change, or protecting major dispersal corridors for plant species from urban development, “Business as usual” urban growth, exemplified by sprawl, was the least favorable scenario for biodiversity (69).

Climate change also interacts with biological invasions to erode biodiversity. Trait-based modeling indicates that future climate change will favor non-native over native grass species in already heavily invaded California grasslands (70). Field experiments have shown that nitrogen deposition, climate change (increasing/decreasing aridity), and plant invasions (71) or elevated CO_2_ and fire (72) interact in complex ways to alter ecosystem processes (e.g., primary productivity, nutrient acquisition by plants) and their drivers (e.g., arbuscular mycorrhizal fungi) in California. Invasive annual grasses have promoted novel fire regimes in California’s deserts with negative ecological consequences, and climate change is expected to accelerate and amplify this grass-fire cycle (73).

There is increasing evidence of secondary climate change effects, in which pests and pathogens respond to changing environmental conditions, as well as to stress in their host species (74). This is particularly evident in tree-dominated ecosystems. The effects of hotter drought over the past decade in California (75) have led cumulatively to mass tree mortality in the Sierra Nevada Mountains. The unprecedented scale of tree mortality, with associated increase in fuel loads and vulnerability to bark beetles, presents an increased risk of large, severe fires in the coming decades (76). Sudden Oak Death (SOD; *Phytophthora ramorum*) is estimated to have killed 48 million trees in coastal and northern California and infected 150 million more since 1995, with 1.8 billion remaining at risk (77). The SOD pathogen benefits from warmer rainy temperatures, and although a direct connection has not been established, historical warming of air temperature in the wet winter months of California’s north coast ecoregion has increased, with mean air temperature warming of 1.33 ± 0.29°F from 1951 to 1980 (33.63°F) to 1981 to 2020 (34.95°F) (78).

In summary, climate change impacts on California’s biodiversity will be exacerbated by the loss of newly suitable habitats to land-use changes; lack of connectivity for reaching newly suitable habitats; and increased pressure from invasive species, pests, and pathogens. Changing fire regimes is an additional stressor that may become even more severe under climate change, and we turn to them in the next section.

## 4. Fire-Driven Impacts of Climate on Biodiversity

**Fig. 2.**
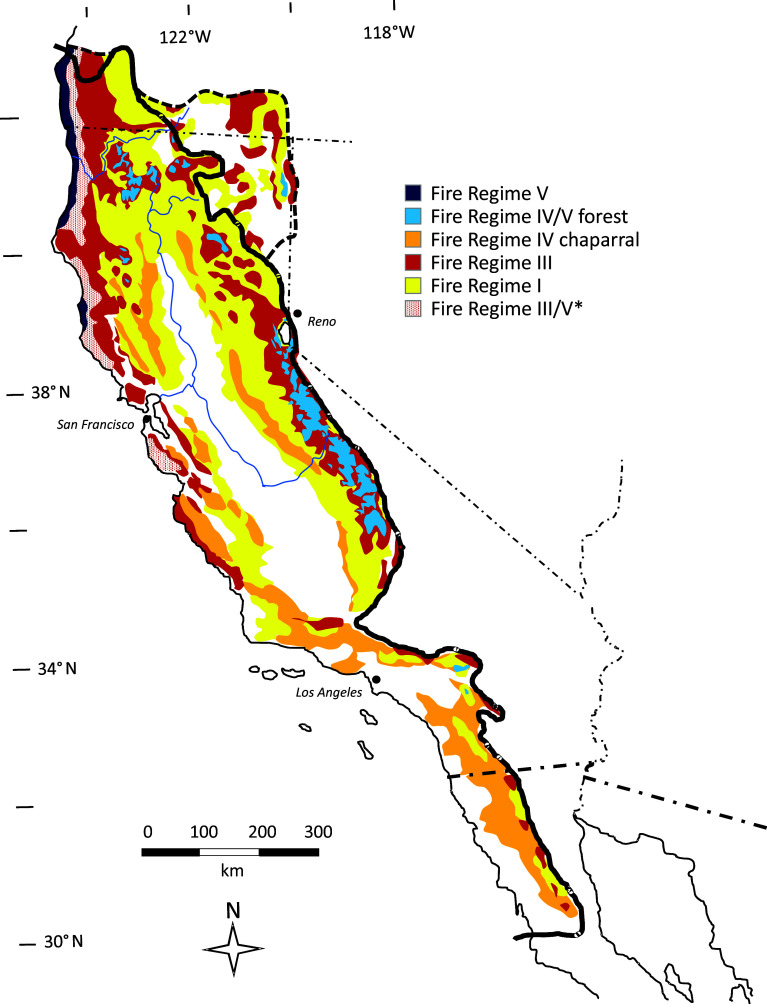
Fire regimes in California, as they existed at the time of the beginning of mass Euro-American settlement in 1850. I—high frequency, low severity; II—high frequency, high severity (not shown); III—moderate frequency, mixed severity; IV—moderate frequency, high severity; V—847 low frequency, high severity. *Redwood (*Sequoia sempervirens*). Figure modified from ref. 80.

Fire has long been a dominant ecological process in California, and California’s diverse fire regimes are strongly shaped by the state’s physical and biotic diversity (79, 80). The term “fire regime” denotes the characteristic frequency, seasonality, extent, and severity of fire at any given place in the landscape. Fire regimes broadly depend on climate, fuels, and ignitions, and over the long term, fire regimes also shape the vegetation that provides the fuel supply. Five major fire regimes (called Fire Regime Groups, or FRG) are recognized by the US Federal Land Management Agencies, ranging in frequency from low (200+ years return interval) to high (1 to 35 y return interval), and in severity from low (<25% of overstory vegetation killed) to high (>75% of overstory vegetation killed). Emblematic of its diversity, the CFP supports all five of these major US fire regimes (Fig. 2). Two fire regimes were historically most prevalent in the state: FRG-I, dominated by high frequency (average of one fire every 5 to 15 y) and low-severity fire, in pine- and oak-dominated lower montane forests and foothill woodlands; and FRG-IV, dominated by moderate frequency (average of one fire every 40 to 90 y) and high-severity fire, in lower elevation chaparral and other shrubland ecosystems. FRG-II, frequent high-severity fire, was once widespread in the state’s valley grasslands, but these have been largely converted to agriculture or urbanization (80).

Recent drastic alterations to long-reigning fire regimes, which have been driven by climate change as well as by more direct human agency, are major drivers of change in terrestrial ecosystem function, structure, and composition in California landscapes (81). The fire season is lengthening at high elevations due to lower snow accumulation, earlier spring snowmelt, and higher summer temperatures, and wildfires have been burning into higher elevations (82). In northern California montane forests, the influence of climate on fire size and annual burned area has increased by twofold to fourfold over the last century (83, 84). At lower elevations, increased temperatures and decreased precipitation are propelling an increase in highly destructive wind-driven autumn wildfires (85). Forest cover has declined by nearly 7% since 1985, almost entirely because of fire (86), and many ecosystems are undergoing type conversion (i.e., conversion to different dominant species or life form) under the influence of unusually frequent and/or severe fire (87).

Forests and woodlands with historically frequent low-severity fire (FRG-I) have been hit by a wave of very large (up to 400,000 ha) and increasingly severe wildfires in the last 20 y, with both climate change and the past century of fire suppression playing critical roles. Forest recovery is imperiled by habitat loss, vegetation type conversion, and low levels of tree regeneration driven partly by increasing climatic moisture deficits (88). Recent studies in California have identified strong negative impacts of high-severity burning and/or large high-severity burn patches in these forests on the diversity of a variety of taxa, including lichens (89), vascular plants (90), large and mesocarnivores (91), and birds (92, 93). Various ecosystem properties and processes are also degraded under high severity burning in these forest types, including old-growth forest, forest structural heterogeneity, carbon storage, and soil biogeochemical cycles (94–96). Essentially, all published models project increasing fire size and severity in these forest types as the 21st century progresses (97).

Chaparral, sage scrub, and sagebrush shrublands with infrequent high-severity fires (FRG-V) are threatened more by increasing fire frequency than severity. Unlike FR- I forests, long-term fire exclusion has not had deleterious effects on these shrublands, and indeed fire suppression may be the only factor preventing large areas of these ecosystems from being irretrievably lost (98). Major threats include frequent human-caused ignitions, nitrogen deposition in and near urban areas, and non-native annual grasses, which enhance fuel loads (98). Climate change impacts on these mostly arid ecosystems and their fire regimes are not as profound as those in more mesic FRG-I forests (99). Nonetheless, rapidly warming temperatures, later arrival of rains, and longer and more severe drought cycles are increasing shrub mortality rates, in turn augmenting fuels and feeding more rapid fire growth (100). In many parts of California, fire frequencies today are so high that the CFP’s iconic and diverse chaparral is hard pressed to persist, while exotic annual grasses have become prevalent.

Fire also interacts with other disturbances to impact ecosystems and biodiversity. The 2012 to 2016 drought and subsequent water stress have provoked major forest dieback in the Sierra Nevada primarily due to pest outbreaks (*Dendroctonus brevicomis, Scolytus ventralis*, among others) in dense, fire-suppressed forest stands. At the same time, the overall trend in California is for much higher variability in annual precipitation, and some of the wettest months and years in history have also occurred in the last decade. Numerous ecologically and economically important forest diseases—e.g., *Phytophthora* spp., *Cronartium ribicola*—are positively linked to precipitation, leading to higher tree mortality in the absence of drought. In either case, increased dead fuel loading from extensive woody plant mortality can increase ember cast, fire spread, and fire intensity, leading to larger fires and more severe fire effects (101, 102).

In summary, under the combined impacts of climate change and other stresses, California’s diverse fire regimes are changing toward larger, more frequent, and/or more severe fires that threaten the resilience of natural vegetation and native species. In the next section, we consider how Californian biodiversity is affected by a key climate change mitigation response: land-intensive renewable energy development.

## 5. Impacts of Renewable Energy Development on Biodiversity

California already hosts more renewable energy than any other state (103), and this capacity is expanding rapidly on land and water to meet the state’s climate change goal of 100% carbon neutrality by 2045 (104, 105). While the substitution of fossil fuels with low-carbon sources of energy is a critical aspect of climate change mitigation, area-based conservation is the primary means for conserving terrestrial biodiversity (106), introducing the potential for conflict between these goals. However, the rapid buildout of new energy infrastructure also creates beneficial opportunities for certain species and ecosystem services (107–110).

For example, at least 72 GW of solar energy capacity (with 37 GW of storage) is anticipated by the state Air Resources Board to be necessary to decarbonize the state’s energy system by 2045. To date, the development of solar energy in California has emphasized large, ground-mounted installations sited predominantly in California’s deserts and the CFP (104). There has been less emphasis on solar energy development within the built environment and other land-sparing recipient environments (110). To support grid integration of these new solar facilities, as well as a quadrupling of wind capacity and 1 GW of new geothermal, state utility experts recommend approximately 50 new transmission projects. Impacts of new energy transmission infrastructure on biodiversity occur during both site preparation and operation and notably include collision- and electrocution-related mortality in birds. Large, ground-mounted solar energy development can affect biodiversity especially strongly during siting and site preparation, which typically includes removing all aboveground vegetation via bulldozing, grading the soil surface, and sometimes constructing large basins to divert and capture runoff (111). Environmental impacts of solar energy development are better documented in California than in most other regions, and include (but are not limited to) effects on land resources (111), land surface temperature (112), soils and hydrology (113), plants (114), insects (115), birds (116), reptiles (117), and mammals (118), In contrast, the impacts of wind energy occur more in during operation; for example, thousands of bird and bat fatalities per year have been documented at the Altamont Pass Wind Resource Area (119, 120).

Energy development that seeks to reduce impacts on biodiversity prioritizes meeting energy demands locally, where generation occurs as close as possible to demand loads (121). The greater the spatial separation between where energy is being generated and where it is being consumed, the greater the risks for adverse consequences on biodiversity and the communities that value it vis-a-vis environmental justice. Hoffacker and Hernandez (122) coined the term “outsiting” as the process by which large, centralized energy infrastructure is relegated to locations beyond the responsible decision-maker’s jurisdictional footprint.

In summary, the potential for adverse effects of renewable energy development on biological conservation is substantial, but there are also opportunities to mitigate these impacts through intentional planning and partnership. The next section considers California’s recent policy commitments aimed at protecting biodiversity in a changing climate.

## 6. Recent Policy Responses and Opportunities

Protecting the unique biological diversity of California’s terrestrial ecosystems requires policies that accommodate the complex interactions of climate change with land-use change, species invasions, changing fire regimes, renewable energy development, and other competing pressures and stressors. Here, we highlight two areas in which California is advancing its climate change adaptation and mitigation plans while maintaining its commitment to protecting biodiversity: its 30 × 30 Initiative and its efforts to mitigate the adverse impacts of renewable energy on biodiversity. We also highlight a third area, fire management, in which policy progress is greatly needed.

### 6.1. California’s 30 × 30 Plan.

Conservation of biodiversity under climate change is the centerpiece of the state’s new “30 × 30 Initiative” or Pathways to 30 × 30: Accelerating Conservation of California’s Nature. California was the first state to commit to the international 30 × 30 movement, under which many countries and now a dozen more US states have committed to “protect biodiversity, advance equitable access to nature and combat climate change” by conserving 30% of their lands and coastal waters by 2030. The California strategy is intended to complement the state’s Natural and Working Lands Climate Smart Strategy and newly launched equity-focused California Outdoors for All Initiative.

The goals of California’s 30 × 30 Initiative most relevant here include: conserving habitats representing the full diversity of California’s ecosystems, especially rare or remnant ones; restoring degraded habitats, especially rare ecosystems; targeting areas for conservation with high species richness, endemism, and rarity; conserving locations that will persist under future climate conditions, serve as refugia for plants and animals, and accommodate habitat range shifts; and, improving habitat connectivity and other actions that build the resilience of species and habitats by facilitating plant and animal migration and gene flow. Actual implementation of the 30 × 30 goals relies on a coordinated effort that began in 2022, led by the California Natural Resources Agency and involving a large array of Federal, State, local, and nonprofit partners. The 30 × 30 plan is strongly information-based, and relies on CA Nature, a set of interactive virtual mapping and visualization tools available to the public, for identifying conservation opportunities and tracking progress.

### 6.2. Reducing Biodiversity Impacts of Renewable Energy.

Aligning a rapid, renewable energy transition with biodiversity conservation requires acknowledging the land resource requirements of various energy infrastructure types (109, 123); understanding and anticipating outcomes of energy-related development on biodiversity; prioritizing land-sparing options and biodiversity-friendly mitigation activities that maximize benefits for biodiversity; and establishing policies and incentives that align these goals. Over a dozen techno-ecological synergies have already been identified (104), such as development of solar energy in the built environment. For example, recent legislation (SB 49) calls for the Department of Transportation to evaluate the potential for using road rights-of-way for renewable energy generation. California is also the site of the first land-sparing floating photovoltaic solar energy (FPV) system in the world, as well as some of the largest existing and proposed FPVs (108).

Another enormous opportunity for the state lies in the potential to stack ecosystem services and restoration with renewable energy development. The California prairie biome, which once characterized the Central Valley, has been reduced in area by 95%. In 2000, California’s nonprofit utility, Sacramento Municipal Utilities District (SMUD), commissioned what was once the largest photovoltaic solar energy parking lot in the world. SMUD is now working alongside UC Davis scientists, the Xerces Society, and the Electric Power Research Institute (EPRI) to restore ecosystem services and prairie habitat at two large, ground-mounted solar energy facilities in the Central Valley. This community-based project seeks to create a model for irrigation-less California prairie restoration under photovoltaic solar energy panels that developers can adopt across the region, especially as some estimate almost 500,000 acres of farmland will be abandoned in the Central Valley to maintain goals for water conservation. Diverse stakeholders are now collaborating to promote the use of abandoned and fallowed cropland for ecological restoration and solar energy across the state (see California Agriculture-Pollinator-Solar Working Group).

### 6.3. Fire Management.

New approaches to managing fire are a critical priority for California. Current policies remain focused on fire suppression despite the inevitability of fire and the ecological necessity for periodic fire in many California ecosystems. In forests and woodlands (FRG-I), stand thinning can alleviate fire hazards and impacts, but it is only practical near human settlements and road infrastructure (124). Greater use of fire as a management and restoration tool may be the only way to mitigate the risks of increasingly catastrophic, stand-replacing fires as the climate warms and fire weather becomes more severe. In these ecosystems, fire has always been a major driver of natural selection and species pool definition, and its long-term absence is leading to dense and vulnerable forests. In chaparral and sage scrub ecosystems (FRG-IV), in contrast, fire suppression is a conservation necessity because of the escalating frequency of anthropogenic fires (98).

Fire management incorporating traditional Indigenous burning practices has been widely discussed as a tool for ecocultural restoration (125–128). For example, the North Fork Mono tribe in northern California have been working with public lands managers to restore montane meadows and oak groves within conifer forests (129), using burning, digging, and other practices that promote traditional foods, materials, and lifeways. However, in comparison with the immense scale of California’s interrelated challenges of fire, climate, and biodiversity protection, the movement toward greater use of Indigenous land-tending practices is likely to have effects that are relatively local in scale.

Fire management policies in California and the western United States, in general, are notably less progressive than policies in the areas of climate and conservation. As in the world’s other Mediterranean climate regions (130), focus in California has been on fire suppression for more than a century, leading to massive accumulations of live and dead biomass in FRG-I forests and woodlands, and ecosystem damage due to, for example, widespread and intensive use of heavy mechanized equipment, large-scale burnout operations, and aerial retardant drops (131). More than 50 y ago, the federal wildfire management agencies made a much-advertised switch from fire suppression to “ecological fire management,” but retrenchment was already underway by the early 2000s (132, 133), and the split between fire management and ecosystem management is arguably as wide today as it has ever been (134, 135).

Recent policy and regulatory efforts have attempted to heal this split. In 2015, State and Federal agencies agreed to increase the use of fire in management, and in 2019, they set ambitious targets for vegetation management and restoration; these targets have gone unmet, however, in part because of frequent bans on the use of prescribed fire due to safety and air quality concerns. National Forests in California are increasingly being zoned for ecological fire use (136). Funding has been made available for vegetation management and fuel reduction through the California Carbon Cap and Trade Market (as of May 2023, cap-and-trade provided $792 million to the California Department of Forestry and Fire Protection for its forest health, forest health research, and forest carbon programs; see California Climate Investments). The 2021 California Wildfire and Forest Resilience Action Plan (137) strongly emphasizes using fire for ecosystem management. Still, economic and political incentives have engendered a deeply entrenched preference for fire suppression that is not easily overcome (130, 132).

We conclude in the sincere hope that California’s current willingness to experiment with bold, knowledge-based solutions to sustaining biodiversity in a changing climate will continue, will be recognized and emulated elsewhere, and will pay off in a better future for natural ecosystems and for humans.

## Supplementary Material

Appendix 01 (PDF)

## Data Availability

All study data are included in the article and/or *SI Appendix*.

## References

[1] J. Franklin, G. M. MacDonald, Climate change and California sustainability –challenges and solutions. Proc. Natl. Acad. Sci. U.S.A., this issue (2024). 10.1073/pnas.2405458121 PMC11317553 39074284

[2] J. Morelli, Environmental sustainability: A definition for environmental professionals. J. Environ. Sustain. **1**, 2–20 (2011).

[3] K. E. Parks, M. Mulligan, On the relationship between a resource based measure of geodiversity and broad scale biodiversity patterns. Biodivers. Conserv. **19**, 2751–2766 (2010).

[4] J. Franklin, H. M. Regan, A. D. Syphard, A framework linking biogeography and species traits to plant species vulnerability under global change in Mediterranean-type ecosystems. Front. Biogeogr. **13**, e51254 (2021).

[5] R. M. Cowling, P. W. Rundel, B. B. Lamont, M. T. K. Arroyo, Plant diversity in Mediterranean-climate regions. Trends Ecol. Evol. **11**, 1035–1046 (1996). 10.1016/0169-5347(96)10044-6 21237880

[6] P. R. Dallman, Plant Life in the World’s Mediterranean Climates. (University of California Press, 1998).

[7] S. Harrison, Plant and Animal Endemism in California (University of California Press, 2013).

[8] M. Power, S. Chandra, P. Gleick, W. E. Gleick, Dietrich, Anticipating responses to climate change and planning for resilience in California’s freshwater ecosystems. Proc. Natl. Acad. Sci. U.S.A., this issue (2024). 10.1073/pnas.2310075121 PMC11317582 39074267

[9] S. Iacobellis, D. R. Cayan, J. T. Abatzoglou, H. Mooney, “Climate” in Ecosystems of California, H. Mooney, E. Zavaleta, Eds. (University of California Press, 2016).

[10] R. C. Graham, A. T. O’Geen, “Soils and geomorphology” in Ecosystems of California, H. Mooney, E. Zavaleta, Eds. (University of California Press, 2016).

[11] C. R. Dolanc, T. Keeler-Wolf, M. G. Barbour, “Vegetation” in Ecosystems of California, H. Mooney, E. Zavaleta, Eds. (University of California Press, 2016).

[12] T. Keeler-Wolf, J. M. Evens, A Manual of California Vegetation (California Native Plant Society, ed. 2, 2009).

[13] J. W. Van WagtendonkEd., Fire in California's Ecosystems (University of California Press, 2018).

[14] J. E. Keeley, H. D. Safford, “Fire as an ecosystem process” in Ecosystems of California, H. Mooney, E. Zavaleta, Eds. (University of California Press, 2016).

[15] T. L. Jones, K. Hadick, “Indigenous California” in Ecosystems of California, H. Mooney, E. Zavaleta, Eds. (University of California Press, 2016).

[16] B. G. Baldwin, Species richness and endemism in the native flora of California. Am. J. Bot. **104**, 487–501 (2017), 10.3732/AJB.1600326. 28341628

[17] D. O. Burge, Plant diversity and endemism in the California Floristic province. Madroño **63**, 3–206 (2016), 10.3120/madr-63-02-3-206.1.

[18] P. H. Raven, D. I. Axelrod, Origin and Relationships of the California Flora (University of California Press, 1978).

[19] C. I. Millar, W. B. Woolfenden, “Ecosystems past: Vegetation prehistory” in Ecosystems of California, H. Mooney, E. Zavaleta, Eds. (University of California Press, 2016).

[20] P. W. Rundel, Fire and plant diversification in Mediterranean-climate regions. Front. Plant Sci. **9**, 851 (2018). 30018621 10.3389/fpls.2018.00851 PMC6038726

[21] B. Tershy, “Biodiversity” in Ecosystems of California, H. Mooney, E. Zavaleta, Eds. (University of California Press, 2016).

[22] D. W. Steadman, N. G. Miller, California condor associated with spruce-jack pine woodland in the late Pleistocene of New York. Q. Res. **28**, 415–426 (1987).

[23] E. Zavaleta, “Biological invasions” in Ecosystems of California, H. Mooney, E. Zavaleta, Eds. (University of California Press, 2016).

[24] P. S. Alagona, T. Paulson, A. B. Esch, J. Marter-Kenyon, “Population and land use” in Ecosystems of California, H. Mooney, E. Zavaleta, Eds. (University of California Press, 2016).

[25] D. L. Hankins, Climate resilience through ecological stewardship. Proc. Natl. Acad. Sci. U.S.A., this issue (2024).

[26] M. Greenberg, H. Angelo, H. Losada, C. C. Wilmers, Relational geographies of urban unsustainability: The entanglement of California’s housing cCrisis with WUI growth and climate change. Proc. Natl. Acad. Sci. U.S.A., this issue (2024). 10.1073/pnas.2310080121 PMC11317566 39074270

[27] J. Medellin-Azuara, A. Escriva-Bou, A. C. M. Gaudin, K. Schwabe, D. A. Sumner, Cultivating climate resilience in California agriculture: Adaptations to an increasingly volatile water future. Proc. Natl. Acad. Sci. U.S.A., this issue (2024). 10.1073/pnas.2310079121 PMC11317594 39074271

[28] S. Pincetl, T. Watt, M. J. Santos, “Land use regulation for environmental conservation” in Ecosystems of California, H. Mooney, E. Zavaleta, Eds. (University of California Press, 2016).

[29] J. H. Thorne, R. M. Boynton, L. E. Flint, A. L. Flint, The magnitude and spatial patterns of historical and future hydrologic change in California’s watersheds. Ecosphere **6**, 1–30 (2015), 10.1890/ES14-00300.1.

[30] J. Franklin, J. M. Serra-Diaz, A. D. Syphard, H. M. Regan, Global change and terrestrial plant community dynamics. Proc. Natl. Acad. Sci. U.S.A. **113**, 3725–3734 (2016). 26929338 10.1073/pnas.1519911113 PMC4833242

[31] C. B. Field, N. R. Chiariello, “Climate change impacts” in Ecosystems of California, H. Mooney, E. Zavaleta, Eds. (University of California Press, 2016).

[32] J. H. Thorne, B. J. Morgan, J. A. Kennedy, Vegetation change over sixty years in the Central Sierra Nevada, California, USA. Madroño **55**, 223–237 (2008).

[33] A. Kelly, M. Goulden, Rapid shifts in plant distribution with recent climate change. Proc. Natl. Acad. Sci. U.S.A. **105**, 11823–11826 (2008). 18697941 10.1073/pnas.0802891105 PMC2575286

[34] A. P. Hill, C. L. Nolan, T. W. Cambron, C. B. Field, Low-elevation conifers in California’s Sierra Nevada are out of equilibrium with climate. PNAS Nexus **2**, pgad004 (2023), 10.1093/pnasnexus/pgad004. 36874277 PMC9976749

[35] P. McIntyre, 20th century shifts in forest structure in California: Denser forests, smaller trees, and increased dominance of oaks. Proc. Natl. Acad. Sci. U.S.A. **112**, 1458–1463 (2015), 10.1073/pnas.1410186112. 25605888 PMC4321274

[36] M. W. Tingley, The push and pull of climate change causes heterogeneous shifts in avian elevational ranges. Global Change Biol. **18**, 3279–3290 (2012).

[37] K. C. Rowe, Spatially heterogeneous impact of climate change on small mammals of montane California. Proc. R. Soc B Biol. Sci. **282**, 20141857 (2015). 10.1098/rspb.2014.1857 PMC4286042 25621330

[38] G. Rapacciuolo, Beyond a warming fingerprint: Individualistic biogeographic responses to heterogeneous climate change in California. Global Change Biol. **20**, 2841–2855 (2014). 10.1111/gcb.12638 PMC4145667 24934878

[39] J. M. Serra-Diaz, California forests show early indications of both range shifts and local persistence under climate change. Global Ecol. Biogeogr. **25**, 164–175 (2016).

[40] C. A. Halsch, A. M. Shapiro, J. Fordyce, M. Forister, Insects and recent climate change. Proc. Natl. Acad. Sci. U.S.A. **118**, e2002543117 (2021). 33431560 10.1073/pnas.2002543117 PMC7812774

[41] C. Boisvenue, S. W. Running, Impacts of climate change on natural forest productivity–evidence since the middle of the 20th century. Global Change Biol. **12**, 862–882 (2006).

[42] S. Harrison, M. J. Spasojevic, D. Li, Inaugural Article: Climate and plant diversity in space and time. Proc. Natl. Acad. Sci. U.S.A. **117**, 4464–4470 (2020). 32071212 10.1073/pnas.1921724117 PMC7060689

[43] S. Harrison, Plant community diversity will decline more than increase under climatic warming. Philos. Trans. R. Soc. B **375**, 20190106 (2020). 10.1098/rstb.2019.0106 PMC7017766 31983333

[44] S. R. Loarie, Climate change and the future of California’s endemic flora. PLoS One **3**, e2502 (2008), 10.1371/journal.pone.0002502. 18648541 PMC2481286

[45] H. Choe, Meta-corridor solutions for climate-vulnerable plant species groups in South Korea. J. Appl. Ecol. **54**, 1742–1754 (2017), 10.1111/1365-2664.12865.

[46] T. L. Morelli, Climate-change refugia: Biodiversity in the slow lane. Front. Ecol. Environ. **18**, 228–234 (2020), 10.1002/FEE.2189. 33424494 PMC7787983

[47] J. M. Cartwright, Oases of the future? Springs as potential hydrologic refugia in drying climates. Front. Ecol. Environ. **18**, 245–253 (2020), 10.1002/FEE.2191.

[48] J. H. Thorne, Vegetation refugia can inform climate-adaptive land management under global warming. Front. Ecol. Environ. **18**, 281–287 (2020), 10.1002/FEE.2208.

[49] H. M. Regan, Evaluation of assisted colonization strategies under global change for a rare, fire-dependent plant. Global Change Biol. **18**, 936–947 (2012).

[50] E. I. Damschen, Endemic plant communities on special soils: Early victims or hardy survivors of climate change? J. Ecol. **100**, 1122–1130 (2012).

[51] J. M. Serra-Diaz, J. Franklin, What’s hot in conservation biogeography in a changing climate? Going beyond species range dynamics. Divers. Distrib. **25**, 492–498 (2019).

[52] J. Franklin, H. M. Regan, A. D. Syphard, A framework linking biogeography and species traits to plant species vulnerability under global change in Mediterranean-type ecosystems. Front. Biogeography **13**, e51254 (2021).

[53] J. Franklin, Moving beyond static species distribution models in support of conservation biogeography. Divers. Distrib. **16**, 321–330 (2010).

[54] A. Staudt, The added complications of climate change: Understanding and managing biodiversity and ecosystems. Front. Ecol. Environ. **11**, 494–501 (2013).

[55] F. W. Davis, Gap analysis of the actual vegetation of California 1. The southwestern region. Madrono **42**, 40–78 (1995).

[56] A. Schoenherr, A Natural History of California (University of California Press, 2017).

[57] S. Pincetl, Transforming California: A Political History of Land Use and Development (JHU Press, 2003).

[58] K. Easterday, P. McIntyre, M. Kelly, Land ownership and 20th century changes to forest structure in California. Forest Ecol. Manag. **422**, 137–146 (2018).

[59] J. E. Lovich, D. Bainbridge, Anthropogenic degradation of the southern California desert ecosystem and prospects for natural recovery and restoration. Environ. Manag. **24**, 309–326 (1999). 10.1007/s002679900235 10486042

[60] K. L. Casner, Contribution of urban expansion and a changing climate to decline of a butterfly fauna. Conserv. Biol. **28**, 773–782 (2014), 10.1111/cobi.12241. 24527888

[61] M. L. Forister, Compounded effects of climate change and habitat alteration shift patterns of butterfly diversity. Proc. Natl. Acad. Sci. U.S.A. **107**, 2088–2092 (2010). 20133854 10.1073/pnas.0909686107 PMC2836664

[62] S. A. MacLean, A. F. Rios Dominguez, P. de Valpine, S. R. Beissinger, A century of climate and land-use change cause species turnover without loss of beta diversity in California’s Central Valley. Global Change Biol. **24**, 5882–5894 (2018). 10.1111/gcb.14458 30267548

[63] K. B. Byrd, Integrated climate and land use change scenarios for California rangeland ecosystem services: Wildlife habitat, soil carbon, and water supply. Landscape Ecol. **30**, 729–750 (2015).

[64] D. M. Lawson, H. M. Regan, P. H. Zedler, J. Franklin, Cumulative effects of land use, altered fire regime and climate change on persistence of *Ceanothus verrucosus*, a rare, fire-dependent plant species. Global Change Biol. **16**, 2518–2529 (2010).

[65] E. Conlisk, Management implications of uncertainty in assessing impacts of multiple landscape-scale threats to species persistence using a linked modeling approach. Global Change Biol. **3**, 858–869 (2013). 10.1111/gcb.12090 23504842

[66] D. Jongsomjit, Between a rock and a hard place: The impacts of climate change and housing development on breeding birds in California. Landscape Ecol. **28**, 187–200 (2013).

[67] E. C. Riordan, P. W. Rundel, Land use compounds habitat losses under projected climate change in a threatened California ecosystem. PloS One **9**, e86487 (2014). 24466116 10.1371/journal.pone.0086487 PMC3897708

[68] M. B. Rose, S. J. E. Velazco, J. Franklin, H. M. Regan, Rarity, geography, and plant exposure to global change in the California Floristic Province. Global Ecol. Biogeogr. **32**, 218–232 (2023).

[69] J. H. Thorne, Infill outperforms climate-adaptive urban growth strategies for regional sustainability. Landscape Urban Plann. **157**, 483–492 (2017), 10.1016/j.landurbplan.2016.08.013.

[70] B. Sandel, E. M. Dangremond, Climate change and the invasion of California by grasses. Global Change Biol. **18**, 277–289 (2012).

[71] S. E. Weber, Responses of arbuscular mycorrhizal fungi to multiple coinciding global change drivers. Fungal Ecol. **40**, 62–71 (2019).

[72] H. A. Henry, Interactive effects of fire, elevated carbon dioxide, nitrogen deposition, and precipitation on a California annual grassland. Ecosystems **9**, 1066–1075 (2006).

[73] J. T. Abatzoglou, C. A. Kolden, Climate change in western US deserts: Potential for increased wildfire and invasive annual grasses. Rangeland Ecol. Manag. **64**, 471–478 (2011).

[74] Z. J. Robbins, Warming increased bark beetle-induced tree mortality by 30% during an extreme drought in California. Global Change Biol. **22**, 509–523 (2022), 10.1111/gcb.15927. 34713535

[75] D. Griffin, K. J. Anchukaitis, How unusual is the 2012–2014 California drought? Geophys. Res. Lett. **41**, 9017–9023 (2014).

[76] S. L. Stephens, Drought, tree mortality, and wildfire in forests adapted to frequent fire. Bioscience **68**, 77–88 (2018).

[77] R. C. Cobb, The magnitude of regional-scale tree mortality caused by the invasive pathogen *Phytophthora ramorum*. Earth’s Future **8**, e2020EF001500 (2020), 10.1029/2020EF001500.

[78] L. E. Flint, A. L. Flint, M. A. Stern, The Basin Characterization Model - A Regional Water Balance Software Package (BCMv8) Data Release and Model Archive for Hydrologic California, Water Years 1896–2020, U.S. (Geological Survey data release, 2021), 10.5066/P9PT36UI.

[79] A. D. Syphard, S. J. E. Velazco, M. B. Rose, H. M. Regan, J. Franklin, The importance of geography in mapping future fire patterns in California, special feature on climate change and california’s sustainability. Proc. Natl. Acad. Sci U.S.A., this issue (2024). 10.1073/pnas.2310076121 PMC11317612 39074287

[80] H. D. Safford, “Fire ecology of the North American Mediterranean-climate zone” in Fire Ecology and Management: Past, Present, and Future of US Forested Ecosystems, B. Collins, C. H. Greenberg, Eds. (Springer, 2021), pp. 337–392.

[81] C. R. Restaino, H. D. Safford, “Fire and climate change” in Fire in California’s Ecosystems, J. Van Wagtendonk, Eds. (University of California Press, ed. 2, 2018), pp. 493–500.

[82] M. W. Schwartz, Increasing elevation of fire in the Sierra Nevada and implications for forest change. Ecosphere **6**, 121 (2015), 10.1890/ES15-00003.1.

[83] J. D. Miller, H. D. Safford, M. Crimmins, A. E. Thode, Quantitative evidence for increasing forest fire severity in the Sierra Nevada and southern Cascade Mountains, California and Nevada, USA. Ecosystems **12**, 16–32 (2009).

[84] J. D. Miller, Trends and causes of severity, size, and number of fires in northwestern California, USA. Ecol. Appl. **22**, 184–203 (2012). 22471083 10.1890/10-2108.1

[85] M. Goss, Climate change is increasing the likelihood of extreme autumn wildfire conditions across California. Environ. Res. Lett. **15**, 094016 (2020).

[86] J. A. Wang, Losses of tree cover in California driven by increasing fire disturbance and climate stress. AGU Adv. **3**, e2021AV000654 (2022).

[87] C. Guiterman, Vegetation type conversion in the US Southwest: Frontline observations and management responses. Fire Ecol. **18**, 6 (2022).

[88] K. T. Davis, Reduced fire severity offers near-term buffer to climate-driven declines in conifer resilience across the western United States. Proc. Natl. Acad. Sci. U.S.A. **120**, e2208120120 (2023). 36877837 10.1073/pnas.2208120120 PMC10089158

[89] J. E. D. Miller, H. Root, H. D. Safford, Altered fire regimes cause long-term lichen diversity losses. Global Change Biol. **24**, 4909–4918 (2018). 10.1111/gcb.14393 30091212

[90] C. J. Richter, The species diversity x fire severity relationship is hump-shaped in yellow pine and mixed conifer forests. Ecosphere **10**, e02882 (2019).

[91] B. J. Furnas, B. R. Goldstein, P. J. Figura, Intermediate fire severity diversity promotes richness of forest carnivores in California. Divers. Distrib. **28**, 493–505 (2022).

[92] A. Kramer, California spotted owl habitat selection in a fire-managed landscape suggests conservation benefit of restoring historical fire regimes. Forest Ecol. Manag. **479**, 118576 (2021).

[93] Z. L. Steel, When bigger isn’t better – implications of large high-severity wildfire patches on avian diversity and community composition. Divers. Distrib. **28**, 439–453 (2021), 10.1111/ddi.1328.

[94] M. P. North, M. D. Hurteau, High-severity wildfire effects on carbon stocks and emissions in fuels in treated and untreated forest. Forest Ecol. Manag. **261**, 1115–1120 (2011).

[95] Z. L. Steel, M. Koontz, H. D. Safford, The changing landscape of wildfire: Burn pattern trends and implications for California’s yellow pine and mixed conifer forests. Landscape Ecol. **33**, 1159–1176 (2018).

[96] N. C. Dove, High-severity wildfire leads to multi-decadal impacts on soil biogeochemistry in mixed conifer forests. Ecol. Appl. **30**, e02072 (2020). 31925848 10.1002/eap.2072

[97] M. H. Dettinger, “Sierra Nevada Summary Report” in California’s Fourth Climate Change Assessment (California Natural Resources Agency and California Energy Commission, publication SUM-CCCA4-2018-004, 2018).

[98] H. D. Safford, E. C. Underwood, N. A. Molinari, “Managing chaparral resources on public lands” in Valuing Chaparral: Ecological, Socioeconomic, and Management Perspectives, E. C. Underwood, H. D. Safford, N. A. Molinari, J. E. Keeley, Eds. (Springer, 2018), pp. 411–448.

[99] J. E. Keeley, A. D. Syphard, Climate change and future fire regimes: Examples from California. Geosciences **6**, 37 (2016).

[100] J. E. Keeley, T. J. Brennan, A. D. Syphard, The effects of prolonged drought on vegetation dieback and megafires in southern California chaparral. Ecosphere **13**, e4203.(2022).

[101] M. R. Metz, K. M. Frangioso, R. K. Meentemeyer, D. M. Rizzo, Interacting disturbances: Wildfire severity affected by stage of forest disease invasion. Ecol. Appl. **21**, 313–320 (2011). 21563563 10.1890/10-0419.1

[102] S. L. Stephens, Mass fire behavior created by extensive tree mortality and high tree density not predicted by operational fire behavior models in the southern Sierra Nevada. Forest Ecol. Manag. **518**, 120258 (2022).

[103] S. Jafarinejad, R. R. Hernandez, S. Bigham, B. S. Beckingham, The intertwined renewable energy–water–environment (REWE) nexus challenges and opportunities: A case study of California. Sustainability **15**, 10672 (2023).

[104] R. R. Hernandez, Solar energy development impacts on land cover change and protected areas. Proc. Natl. Acad. Sci. U.S.A. **112**, 13579–13584 (2015). 26483467 10.1073/pnas.1517656112 PMC4640750

[105] A. E. Cagle, The land sparing, water surface use efficiency, and water surface transformation of floating photovoltaic solar energy installations. Sustainability **12**, 8154 (2020).

[106] S. L. Maxwell, Area-based conservation in the twenty-first century. Nature **586**, 217–227 (2020). 33028996 10.1038/s41586-020-2773-z

[107] R. R. Hernandez, Techno–ecological synergies of solar energy for global sustainability. Nat. Sustain. **2**, 560–568 (2019).

[108] B. McKuin, Energy and water co-benefits from covering canals with solar panels. Nat. Sustain. **4**, 609–617 (2021), 10.1038/s41893-021-00693-8.

[109] B. Turley, Emergent landscapes of renewable energy storage: Considering just transitions in the Western United States. Energy Res. Soc. Sci. **90**, 102583 (2022).

[110] M. K. Hoffacker, M. F. Allen, R. R. Hernandez, Land-sparing opportunities for solar energy development in agricultural landscapes: A case study of the Great Central Valley, CA. United States. Environ. Sci. Technol. **51**, 14472–14482 (2017). 29254337 10.1021/acs.est.7b05110

[111] S. Rangarajan, S. M. Jordaan, R. R. Hernandez, Life cycle impacts of concentrated solar power generation on land resources and soil carbon losses in the United States. Front. Sustain. **3**, 1021971 (2022), 10.3389/frsus.2022.1021971.

[112] L. Guoqing, Ground-mounted photovoltaic solar parks promote land surface cool islands in arid ecosystems. Renew. Sustain. Energy Trans. **1**, 100008 (2021).

[113] K. E. Tanner, Simulated solar panels create altered microhabitats in desert landforms. Ecosphere **11**, e03089 (2020).

[114] S. M. Grodsky, R. R. Hernandez, Reduced ecosystem services of desert plants from ground-mounted solar energy development. Nat. Sustain. **3**, 1036–1043 (2020).

[115] S. M. Grodsky, J. W. Campbell, R. R. Hernandez, Solar energy development impacts flower-visiting beetles and flies in the Mojave Desert. Biol. Conserv. **263**, 109336 (2021).

[116] T. J. Conkling, Vulnerability of avian populations to renewable energy production. R. Soc. Open Sci. **9**, 211558 (2022). 35360356 10.1098/rsos.211558 PMC8965424

[117] D. R. Cameron, B. S. Cohen, S. A. Morrison, An approach to enhance the conservation-compatibility of solar energy development. PloS One **7**, e38437 (2012). 22685568 10.1371/journal.pone.0038437 PMC3369905

[118] R. D. Inman, Impacts of climate change and renewable energy development on habitat of an endemic squirrel, *Xerospermophilus mohavensis*, in the Mojave Desert, USA. Biol. Conserv. **200**, 112–121 (2016).

[119] K. S. Smallwood, Burrowing owl mortality in the Altamont Pass wind resource area. J. Wildlife Manag. **71**, 1513–1524 (2007).

[120] K. S. Smallwood, C. G. Thelander, Bird mortality in the Altamont Pass. J. Wildlife Manag. **72**, 215–223 (2008).

[121] K. Surana, S. M. Jordaan, The climate mitigation opportunity behind global power transmission and distribution. Nat. Climate Change **9**, 660–665 (2019).

[122] M. K. Hoffacker, R. R. Hernandez, Local energy: Spatial proximity of energy providers to their power resources. Front. Sustain. **1**, 585110 (2020).

[123] J. Lovering, M. Swain, L. Blomqvist, R. R. Hernandez, Land-use intensity of electricity production and tomorrow’s energy landscape. PLoS One **17**, e0270155 (2022). 35793381 10.1371/journal.pone.0270155 PMC9258890

[124] M. P. North, Constraints on mechanized treatment significantly limit mechanical fuels reduction extent in the Sierra Nevada. J. For. **113**, 40–48 (2015).

[125] R. W. Kimmerer, F. K. Lake, The role of indigenous burning in land management. J. For. **99**, 36–41 (2001).

[126] K. Anderson, Tending the Wild: Native American Knowledge and the Management of California’s Natural Resources (University of California Press, 2005).

[127] F. K. Lake, Returning fire to the land: Celebrating traditional knowledge and fire. J. For. **115**, 343–353 (2017).

[128] V. Reyes-García, The contributions of Indigenous peoples and local communities to ecological restoration. Restor. Ecol. **27**, 3–8 (2019).

[129] J. W. Long, F. K. Lake, Escaping social-ecological traps through tribal stewardship on national forest lands in the Pacific Northwest, United States of America. Ecol. Soc. **23**, 14 (2018), 10.5751/ES-10041-230210.

[130] F. Moreira, Wildfire management in Mediterranean-type regions: Paradigm change needed. Environ. Res. Lett. **15**, 011001 (2020).

[131] D. M. Backer, S. E. Jensen, G. R. McPherson, Impacts of fire-suppression activities on natural communities. Conserv. Biol. **18**, 937–939 (2004).

[132] S. L. Stephens, L. W. Ruth, Federal forest-fire policy in the United States. Ecol. Appl. **15**, 532–542 (2005).

[133] S. Botti, T. Nichols, National Park Service fire restoration, policies versus results: What went wrong. Parks Stewardship Forum **37**, 353–367 (2021).

[134] M. P. North, Reform forest fire management. Science **349**, 1280–1281 (2015). 26383934 10.1126/science.aab2356

[135] H. D. Safford, A. K. Paulson, Z. L. Steel, D. J. N. Young, R. B. Wayman, The 2020 California fire season: A year like no other, a return to the past, or a harbinger of the future? Global Ecol. Biogeogr. **31**, 2005–2025 (2022), 10.1111/geb.13498.

[136] USDA, 36 CFR 219. National Forest System Land Management Planning. Federal Register **77**, Number 68 (2012).

[137] State of California, California’s Wildfire and Forest Resilience Action Plan (California Forest Management Task Force, 2021), https://fmtf.fire.ca.gov/.

