# Supplementary material for: Climate change and California’s terrestrial biodiversity
Source: Proc Natl Acad Sci U S A. 2024 Jul 29;121(32):e2310074121. doi: 10.1073/pnas.2310074121 (PMC11317609; doi:10.1073/pnas.2310074121)
Supplement: Supplementary file 1 — Appendix 01 (PDF) [file pnas.2310074121.sapp.pdf]

Climate Change and California's Terrestrial Biodiversity – *Final Version*

Susan Harrison, Janet Franklin, Rebecca R. Hernandez, Makihiko Ikegami, Hugh D. Safford, James H. Thorne

Supplemental Materials

Materials and Methods

Species data

We extracted CFP/CA state endemic plant name lists from CalFlora (https://www.calflora.org/; access May 2017). CalFlora covers almost all known plants found in the area, with over 13,000 species, subspecies, varieties and forms (taxa) listed and about 10,000 taxon as native in California. Among these, 2,975 taxa are endemic or near endemic to California (distribution is mostly limited in California according to CalFlora) and 1230 taxa are categorized as rare, endangered or threatened. We obtained additional distribution data for those species from the Consortium of California Herbaria (https://www.cch2.org/portal/; access May 2017). In addition, we also used occurrence data compiled from various surveys and plant survey relevé data (1-3), and GBIF (https://www.gbif.org/; access May 2018). After removing duplicate records, we obtained 4,244,094 occurrence records for 10,102 taxa over California and surrounding states. Over 95% of the record dates are from after 1910, with the majority falling between 1930 and 2010.

Herbarium collection data can be spatially biased because sampling may be influenced by factors such as road and foot-path access, which can lead to over-representation of certain areas or habitats (4). To mitigate, we randomly selected one record per specie per 2.5 arc min grid (~20.25 km2). We then excluded species with less than 10 data points to avoid model overfitting due to low replicates. Since we had six bioclimatic variables and used 70% of occurrence data for model building, we set a cutoff of 10 records so that the number of explanatory variables is lower than the minimum number of occurrence

points. Finally, we modeled current and future ranges of 6418 native plant species and subspecies and varieties, using 655,893 observation records (Figure S1).

Climate data

Projections of future climate were obtained from transient simulations of 14 GCMs (BCC CSM1-1, CCSM4, CNRM-CM5, GFDL-CM3, GISS-E2-R, HadGEM2-AO, HadGEM2-ES, IPSL-CM5A-LR, MIROC-ESM-CHEM, MIROC-ESM,MIROC5, MPI-ESM-LR, MRI CGCM3, and NorESM1-M) available from WorldClim, along with current climate (WorldClim ver 1.4) (https://www.worldclim.org/data/v1.4/worldclim14.html; access in 2017 July).
All models were implemented using the time periods provided by WorldClim; a baseline time period of 1960-1990 and under the CMIP5 RCP8.5 emission scenario for a 20-year period ending in 2080 (average for 2061-2080). Monthly climate data for monthly maximum and minimum temperature and precipitation data at 2.5 Arc minutes were downloaded and bioclim variables (5) generated. We chose six climatic predictor variables for the SDMs, including annual maximum and minimum temperature, temperature seasonality, precipitation of the warmest and coldest quarter, and an aridity index developed by Ikegami and Jenkins (6).

MaxEnt (version 3.4.0.) was used to calculate climate suitability under current and future climates. We used MaxEnt because of its high performance for small number of observation points, as well as its capacity for large climate layers (7). Occurrence data were randomly separated during model building, with 70% used to train each model and 30% for testing. This process was repeated 10 times for each species, and model accuracy scores and predictions calculated.

Analysis

To produce binary range maps from MaxEnt outputs, the logistic threshold for equal sensitivity and specificity was calculated from the test dataset. To evaluate the conservation values of each grid, we calculated the number of species in each grid as well as the sum of range size rarity values for each species (8). To quantify range size rarity, we calculated the inverse of the area of the predicted presence for each species. If a species is predicted to have a larger range, its conservation priority is lower. After calculating species richness and range size rarity for all grid cells, we normalized the values for each variable to a scale of 0 to 1. We then added the two values to develop a range-richness index. The grids with the highest 2% of range-richness values were selected. To recognize individual regional hotspots, we checked their connectivity to other high-scoring areas. We defined clusters using the 4-neighbourhood rule, when the central grid is connected to others on the cardinal axes, but not the diagonal. Then we calculated the area size (i.e., number of grid cells) of each cluster and defined a regional hotspot as a cluster containing more than 11 grid cells.

We calculated regional hotspots for the baseline time period, and for each of the 14 GCMs by 2080 (Figure S2). We combined the outputs of the 14 GCM hotspot models to identify the consensus future hotspots used to compare to those in the baseline time period.

SI Literature Cited

1. CA Department of Fish and Wildlife Vegetation Survey Points, https://gis.data.ca.gov/datasets/CDFW::vegetation-survey-points-cdfw-ds1020-2/explore, Accessed May 2017. (2023)

2. C. Seo C, J. H. Thorne, L. Hannah, W. Thuiller, Scale effects in species distribution models: implications for conservation planning under climate change. Biology Letters 5:39–43. https://doi.org/10.1098/rsbl.2008.0476 (2009)

3. J. Franklin, F. W. Davis, M. Ikegami, A. D. Syphard, L. E. Flint, A. L. Flint, L. Hannah, Modeling plant species distributions under future climates: How fine scale do climate projections need to be? Global Change Biology 19:473–483. https://doi.org/10.1111/gcb.12051 (2013)

4. B. H. Daru, *et al*., Widespread sampling biases in herbaria revealed from large-scale digitization. New Phytologist 217:939–955. https://doi.org/10.1111/NPH.14855 (2018)

5. R. J. Hijmans, S. E. Cameron, J. L. Parra, P. G. Jones, A. Jarvis, Very high resolution interpolated climate surfaces for global land areas. International Journal of Climatology 25:1965–1978. https://doi.org/10.1002/joc.1276 (2005)

6. M. Ikegami, T. A. R. Jenkins, Estimate global risks of a forest disease under current and future climates using species distribution model and simple thermal model – Pine Wilt disease as a model case. Forest Ecology and Management 409:343–352. https://doi.org/10.1016/J.FORECO.2017.11.005 (2018)

7. S. J. Phillips, M. Dudík, Modeling of species distribution with Maxent: new extensions and a comprehensive evaluation. Ecography 31:161–175. https://doi.org/10.1111/j.2007.0906-7590.05203.x (2008)

8. W. Jetz, C. Rahbek, R. K. Colwell, The coincidence of rarity and richness and the potential signature of history in centres of endemism. Ecology Letters 7:1180–1191. https://doi.org/10.1111/j.1461-0248.2004.00678.x (2004)

Table S1. The 15 current Regional Hotspots with area size under current and future conditions (based on the sum model), locations with longitude and latitude, the spatial dynamics exhibited (mode), and the extent of overlap between current and projected future extents (an average from the 14 GCMs, SD, minimum and maximum overlap). For locations, see figure 1.

ID	Regional Hotspot Name	Area (KM2)	Future remnant (KM 2)	Centroid Long/Lat	Mode	Averages in 14 GCM	SD	Max 14 GCMs	Min 14 GCMs
1	Northern Sierra	113	54	-121.32, 39.77	Ecotonal	47.1	21.7	78	0
2	Point Arena - Coast Redwoods	74	36	-123.58, 38.97	Contract	28.6	30.6	74	0
3	Snow Mountain	87	22	-122.58, 39.24	Dissolve	27.2	24.1	86	0
4	Blue Ridge-Berryessa	23	0	-122.55, 38.87	Dissolve	2.5	6.3	23	0
5	Inner Coast Range North	31	4	-121.66, 37.35	Dissolve	7.6	10.6	31	0
6	Central and Southern Coastal Ranges	1643	1109	-120.98, 36.06	Contract	838.3	344.2	1463	202
7	Southern Sierra Nevada Mountains	896	786	-119.0, 36.91	Ecotonal	741.9	70.6	841	584
8	New Idria-Pinnacles	26	25	-120.67, 36.33	Stable	18.4	8.0	26	7
9	Greenhorn Mountains	28	27	-118.36, 35.46	Ecotonal	25.4	3.8	28	17
10	Transverse Ranges	25	24	-118.56, 34.99	Ecotonal	21.4	4.7	25	10
11	Liebre Mountains	19	6	-118.62, 34.68	Ecotonal	8.1	7.1	19	0
12	San Gabriel Mountains	123	109	-117.83, 34.32	Contract	89.5	28.9	120	13
13	San Bernardino Mountains	13	11	-117.06, 34.27	Stable	6.6	5.7	13	0

14	Santa Cruz Island	18	17	-119.75, 34.02	Stable	9.3	7.7	18	0
15	Santa Rosa Island	17	17	-120.1, 33.97	Stable	11.6	6.4	17	
	(Fragmented)*	154	84	-120.17, 36.65		73.0	24.9	108	22

*Fragmented: Sum of all fragmented clusters smaller than 160 km2

Table S2. The 15 current Regional Hotspots with the number of records of native species used, the number predicted under current and future climates by 2080 (Average of 14 GCMs under RCP8.5) within the current range, species richness for the modeled times, and percentage change for the modeled times. For locations, see Figure 1.

ID	Regional Hotspot Name	Observed Species Richness	Predicted Species Richness 1960-1990	Predicted Future Species Richness 2060-2080	Species Richness Change	% Change
1	Northern Sierra	1017	3276	2561	715	-21.80%
2	Point Arena - Coast Redwoods	650	2407	1942	465	-19.30%
3	Snow Mountain	1017	3377	2695	682	-20.20%
4	Blue Ridge-Berryessa	374	2208	1726	482	-21.80%
5	Inner Coast Range North	448	2038	1649	389	-19.10%
6	Central and Southern Coastal Ranges	2418	4487	3793	694	-15.50%
7	Southern Sierra Nevada Mountains	2465	4261	4078	183	-4.30%
8	New Idria-Pinnacles	369	2630	2036	594	-22.60%
9	Greenhorn Mountains	386	3541	2839	702	-19.80%
10	Transverse Ranges	443	3432	2697	735	-21.40%
11	Liebre Mountains	423	2379	1862	517	-21.70%
12	San Gabriel Mountains	1349	3828	3121	708	-18.50%
13	San Bernardino Mountains	637	2907	2071	836	-28.80%
14	Santa Cruz Island	488	2047	1714	333	-16.30%

15	Santa Rosa Island	354	2154	1715	439	-20.40%
	Fragmented units	1782	4726	4285	441	-9.30%

Table S3. The nine emerging Regional Hotspots using the sum of 14 GCMs by 2080 under the RCP8.5 scenario, with area size km2, projected future species richness and centroid longitude and latitude. For locations, see Figure 1.

ID	Spots Name	Size (KM2)	Predicted Future Species Richness 2060-2080	Centroid Longitude/ Latitude
N1	Siskiyou Mountains	14	3500	-123.05, 41.91
N2	Klamath-Shasta-Trinity Mountains	203	3930	-122.84, 41.3
N3	Yollo Bolly Mountains	19	3272	-122.96, 40.05
N4	Sanhedrin Wilderness	14	3970	-122.96, 39.58
N5	Black Butte	13	3644	-122.82, 39.64
N6	Verdi-Anderson Peaks	13	3055	-120.1, 39.1
N7	Desolation Wilderness	20	3174	-120.28, 38.92
N8	Mokelumne Wilderness	18	3313	-120.15, 38.57
N9	Scodie Mountains	14	3829	-118.12,35.58
Fragmented units		225	7306	-121.12, 38.01

Figure S1. Schematic flow chart of calculating regional hotspots. The rules for each step are shown to the left of the actions shown in the boxes. The top row of boxes and the Climate Data box show the input data.

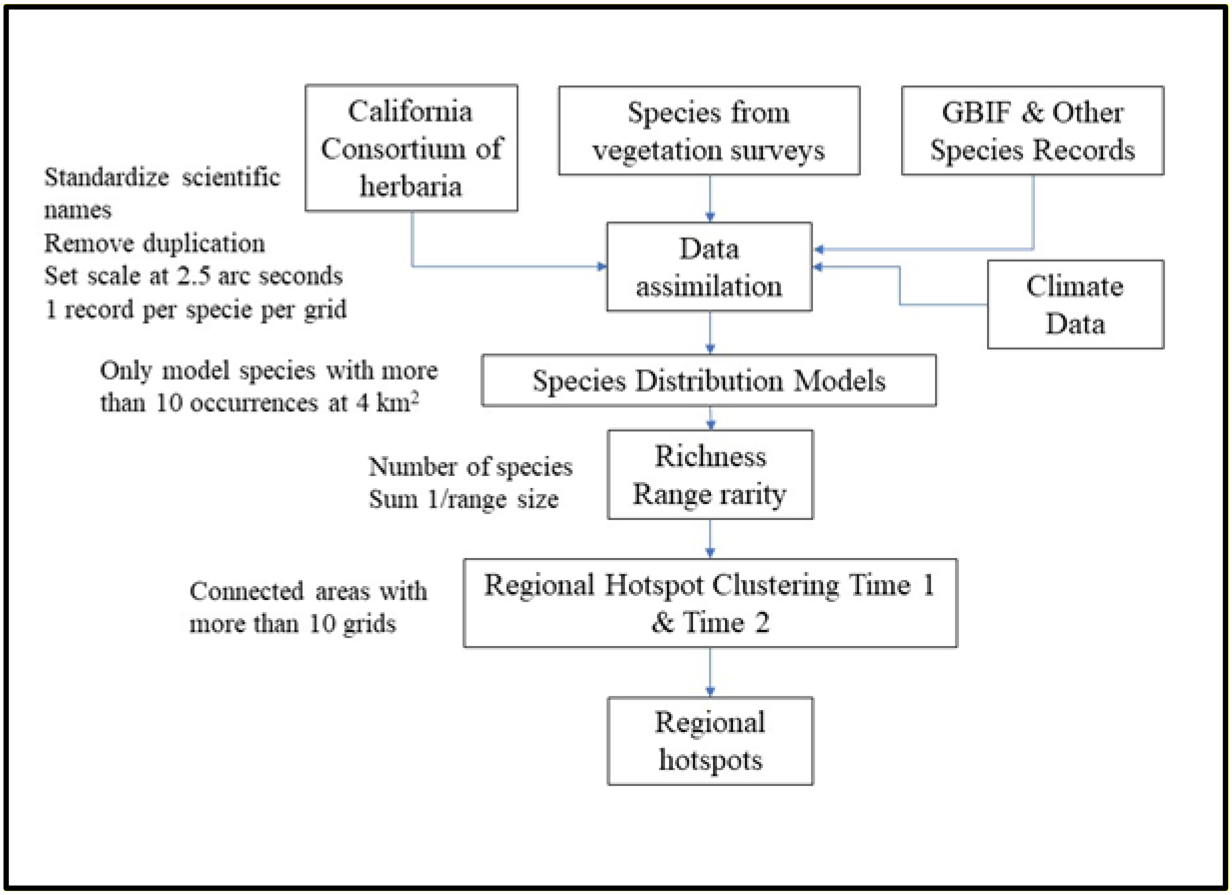

Figure S2. Regional hotspot locations under the baseline time period, as the sum of future projections, and for the 14 individual GCM projections. The sum of future projections is presented in the main text (Figure 1). Some individual GCM projections, such as BCC-CSM2-MR RCP8.5 2060-2080, identify new climatically suitable locations in Oregon and Washington, 100s of kilometers N of the current regional hotspots. These climate windows represent projected suitable climate conditions for a set of species from one of the regional hotspots in California. For example, BCC RCP8.5 2060-2080, projects a hotspot in south-central Washington that contains suitable climatic conditions for an average 1970 taxa from regional hotspot 1.

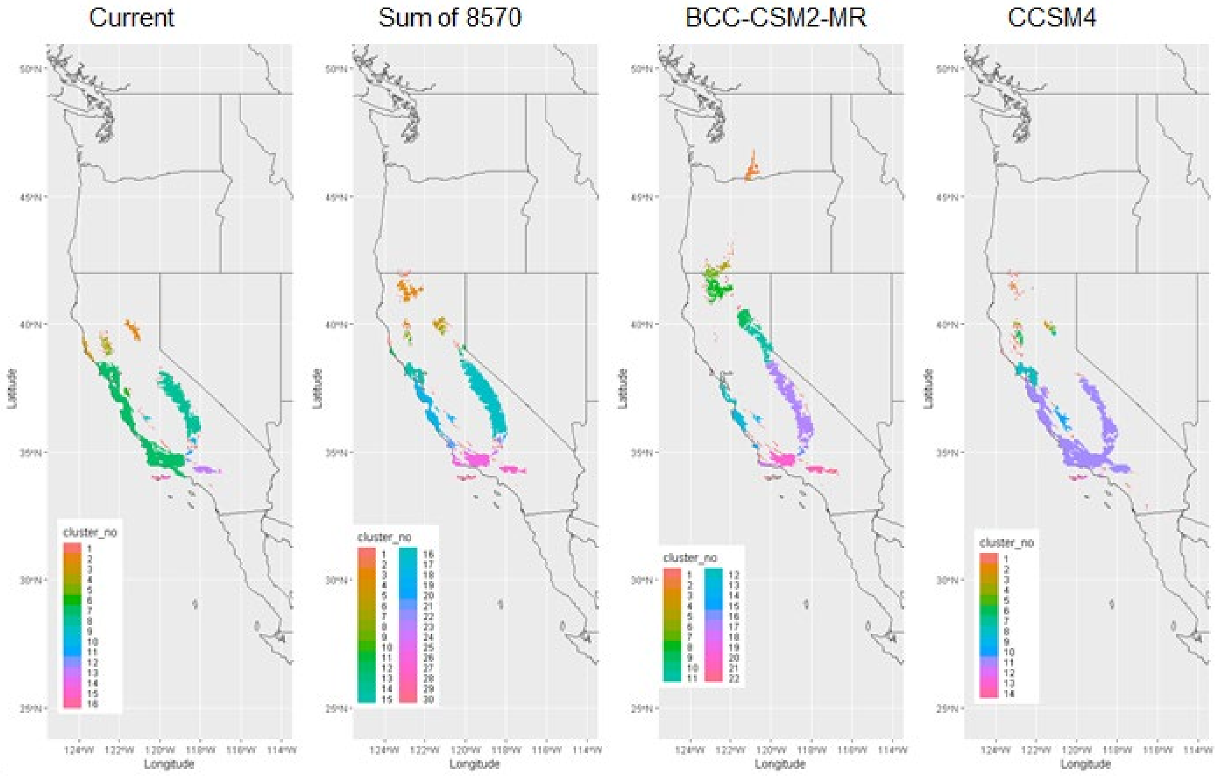

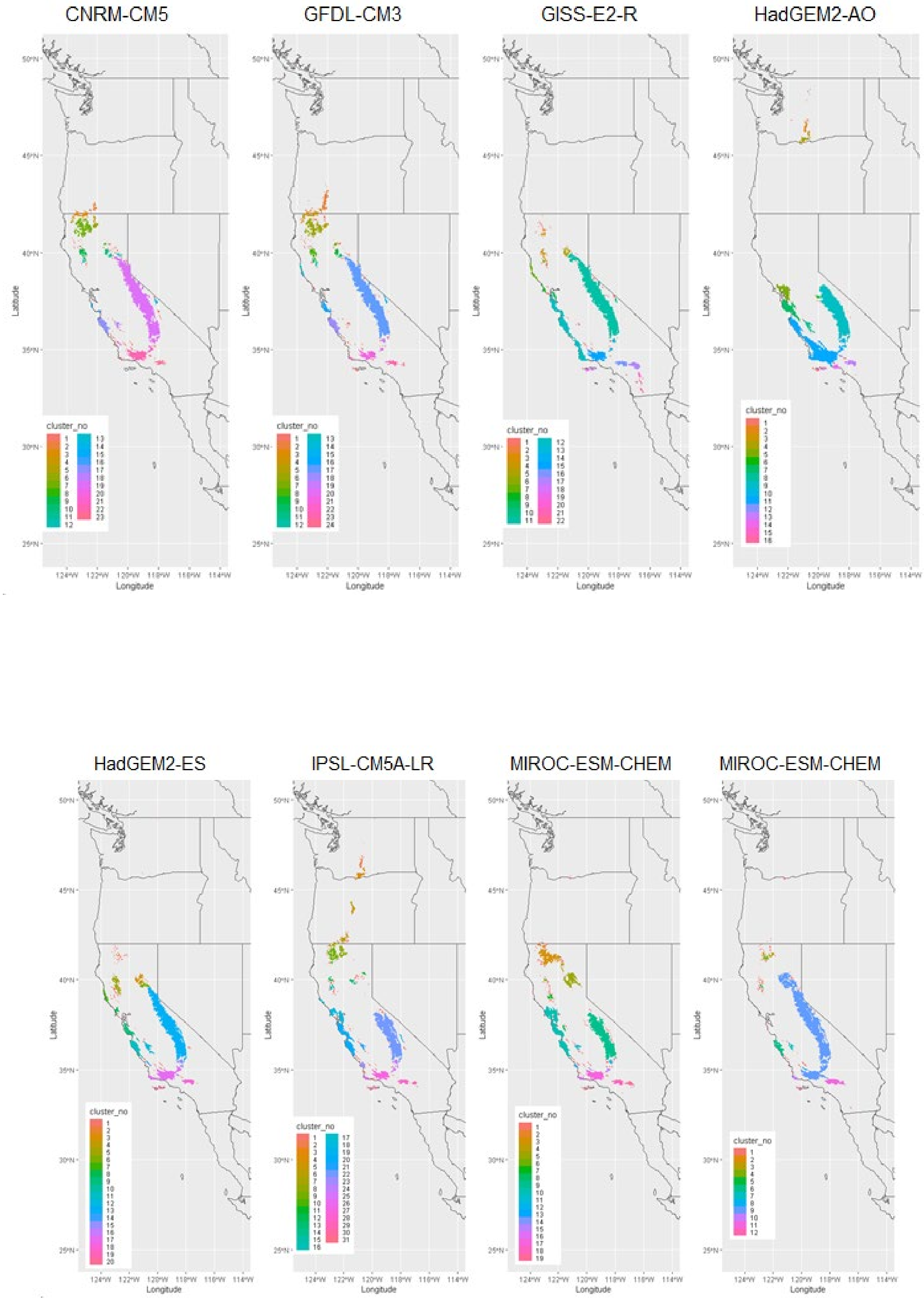
CNRM-CM5
GFDL-CM3
GISS-E2-R
HadGEM2-AO
HadGEM2-ES
IPSL-CM5A-LR
MIROC-ESM-CHEM
MIROC-ESM-CHEM
Latitude
Longitude
cluster_no

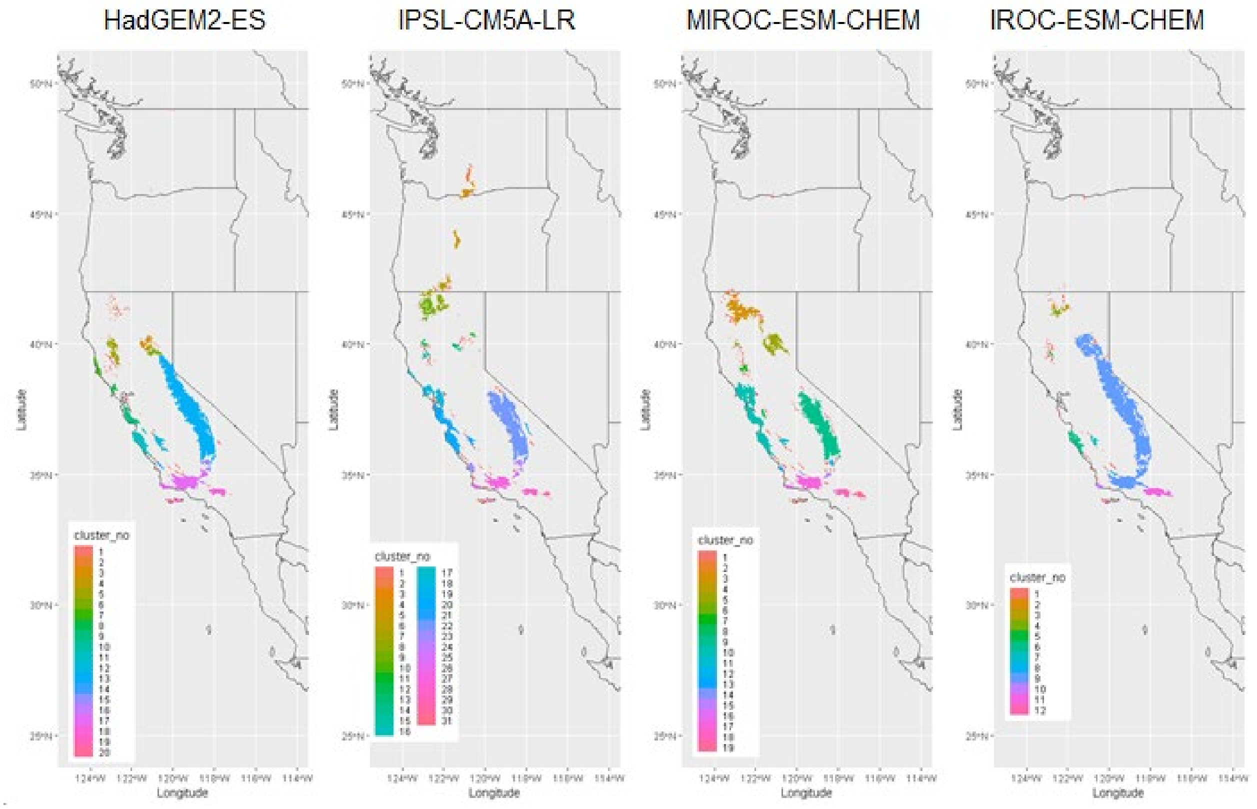
HadGEM2-ES
IPSL-CM5A-LR
MIROC-ESM-CHEM
IROC-ESM-CHEM
Latitude
Longitude
cluster_no
